# Supplementary material for: A large planetary body inferred from diamond inclusions in a ureilite meteorite
Source: Nat Commun. 2018 Apr 17;9:1327. doi: 10.1038/s41467-018-03808-6 (PMC5904174; doi:10.1038/s41467-018-03808-6)
Supplement: Supplementary file 1 — Supplementary Information [file 41467_2018_3808_MOESM1_ESM.pdf]

Supplementary Information

for:

A large planetary body inferred from diamond inclusions in a ureilite meteorite

Nabiei et al.

Supplementary Note 1: Twinning and graphitization induced by the shock

The diamonds in all five samples were highly deformed. This can be inferred from the large population of dislocations, stacking faults and deformation twin throughout the diamond grains (Supplementary Fig. 1 and 2). Stacking faults and twin boundaries are formed along the {111} planes as expected from cubic diamond structure (Supplementary Fig. 1d). The distinct orientation and morphology of twins indicate that they are deformation twins rather than growth or annealing twins (Supplementary Fig. 1c). Moreover, these deformation features point to high strain rates that are consistent with the one expected from a shock event.

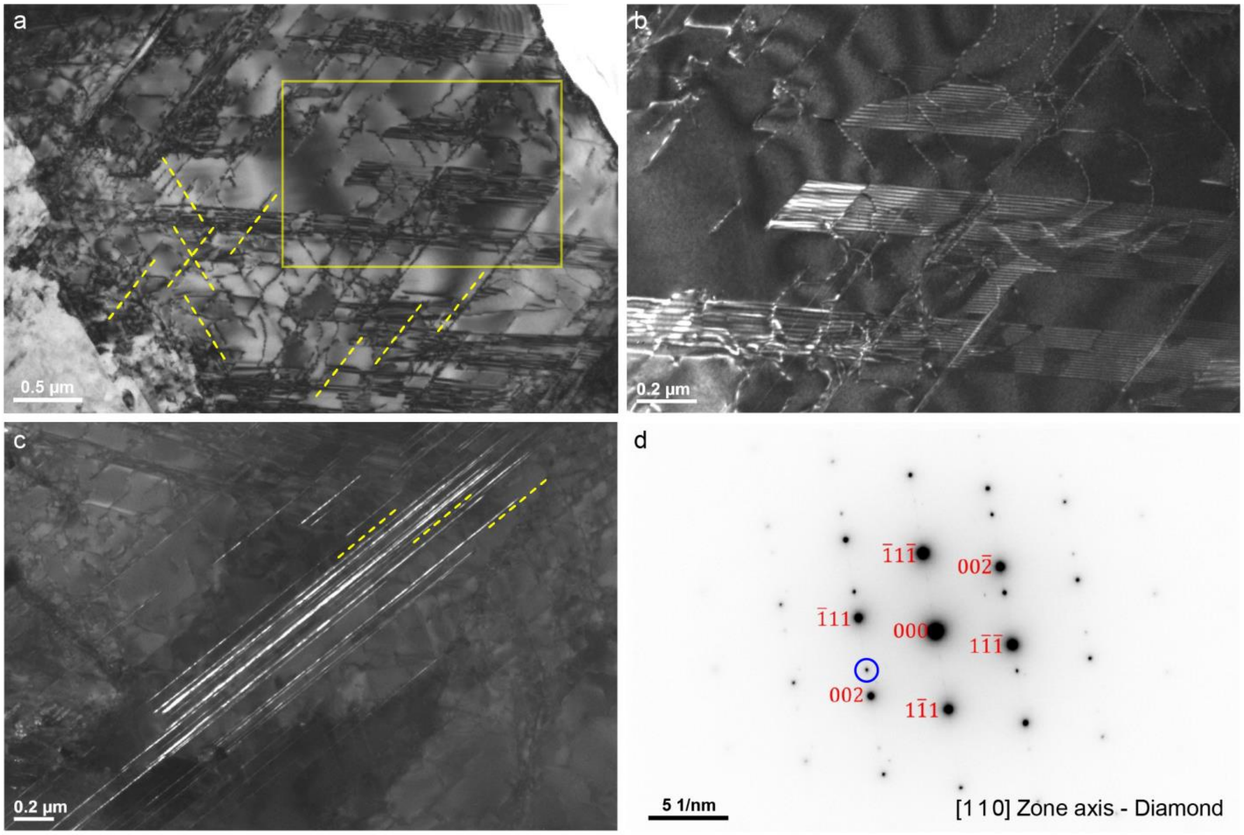

Supplementary Figure 1- a) Bright field (BF) TEM image of the diamond grain oriented in the [110] zone axis. Dashed lines show the trace of {111} planes which are parallel to the optical axis. b) Weak-beam dark field (WBDF) TEM image of dislocations and {111} stacking faults in diamond from the region indicated with yellow rectangle in panel a. c) dark field (DF) TEM image using the $(1\bar{1}\bar{1})$ plane reflection of deformation twins (indicated with blue circle in panel d). d) selected area electron diffraction (SAED) from the [110] zone axis in diamond with indexed diamond reflection (along with mirror reflection for twins).

Analysis of the deformation twins with electron energy loss spectroscopy (EELS) under magic angle condition shows that the carbon generally has sp^3 hybridization consistent with diamond (Supplementary Fig. 2d). However, where specific deformation twins cut through inclusions in diamond they are graphitized and carbon atoms have sp^2 hybridization (Fig. 1 and Supplementary Fig. 2). Supplementary Figure 2 shows two close deformation {111} twins in the diamond. The

one which cuts through the inclusions is graphitized, whereas the other one has no sign of graphitization. Dark field imaging using the (111) plane reflection in twinned crystals shows that the twining front propagates in the diamond matrix until it reaches the inclusions where it graphitizes. In these cases the graphite band keeps the morphology of the original deformation twin. Moreover, while two sides of the graphitized band are still connected they have ~1 degree difference in their crystallographic orientation. This is due to the volume change occurring during the diamond to graphite phase transformation which induces significant strains in the diamond matrix. We have also observed the nucleation of graphite around inclusions in highly deformed regions of diamond (Fig. 4).

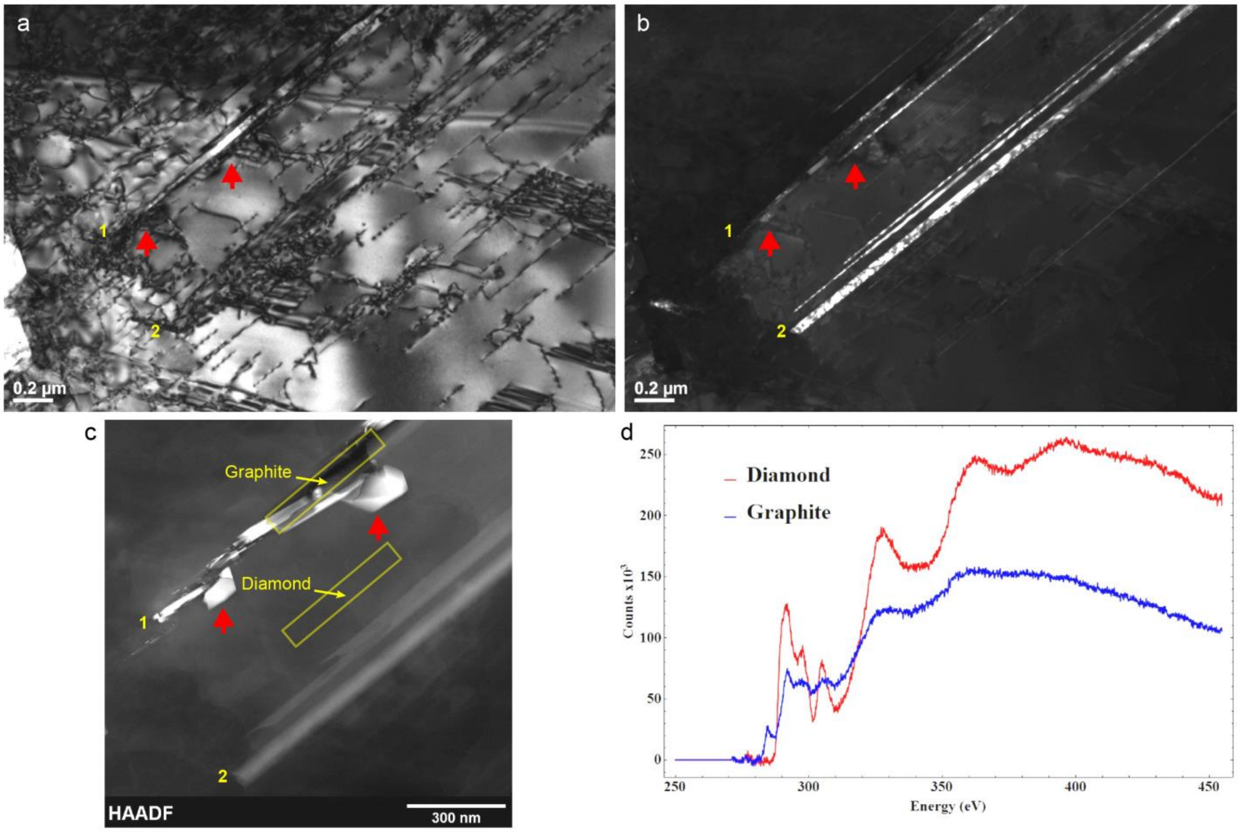

Supplementary Figure 2- a) BF TEM image of a diamond grain oriented in the [110] zone axis shows two twinning regions indicated as "1" and "2". The red arrows indicate the inclusions intersecting with twin number "1". The graphitized region along this twin shows very bright contrast in BF image. b) DF TEM image using the $(1\bar{1}\bar{1})$ plane reflection of deformation twins. The left twin (number "1") transformed to graphite. c) STEM HAADF image of twinning regions. Yellow rectangles show the collection area of EEL spectra reported in panel d. d) Carbon K-edge EEL spectra from diamond (red) and graphite (blue).

Interestingly, the graphite-diamond grain boundaries for a large graphite band separating diamond segments are also parallel to diamond {111} planes (Supplementary Fig. 3). Diamond segments in two sides of these bands have a 1 to 2.5 degree of misorientation with respect to one another. These graphite bands are always filled with inclusions. Therefore, it is plausible to posit

that the large diamond grains existed prior to the shock, and were later cut by graphitization along the deformation twins.

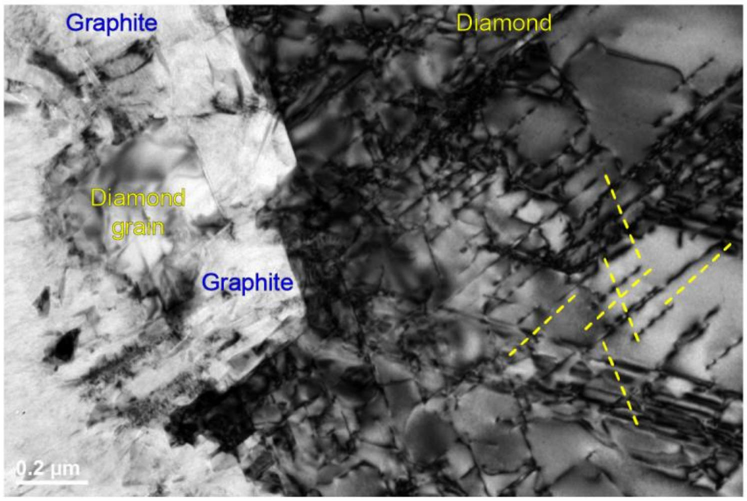

Supplementary Figure 3- BF TEM image of the diamond segment oriented in [110] zone axis indicates diamond-graphite grain boundary with a kinked step-like shape. The grain boundary stays parallel to {111} plains in diamond structure. Dashed lines indicate the trace of {111} planes parallel to the optical axis. We can also see a separated small diamond grain on the left which shows ~40 mrad (~2.3 degree) misorientation with respect to the main grain.

Supplementary Note 2: Iron-sulfur inclusions

Each Fe-S-bearing inclusion consists mainly of metallic and sulfide parts (Fig. 2, Supplementary Fig. 4). The metallic phase is iron with 5.5 atomic% Ni in average (ranging from 4 atomic% to 7 atomic%). Electron nano-diffraction indicates the BCC structure specific to kamacite (Supplementary Fig. 5). The sulfide phase has the composition 50.4 atomic% Fe and 49.6 atomic% S and was characterized as troilite (FeS) by electron diffraction (Supplementary Fig. 5). Moreover, we observed a minor phase with the composition 52.2 atomic% Fe, 22.3% Ni, and 25.4% P which corresponds to schreibersite; the phase was easily distinguishable and individualized in larger grains (Supplementary Fig. 4), but smaller inclusions only exhibit P and Ni enrichments at the interfaces between the diamond matrix and the inclusion, or between troilite and kamacite within the inclusions (Supplementary Fig. 4). We have also detected Cr in these inclusion which is always below 1 atomic% (typically 0.1-0.3 atomic%). Chromium also enriches in the grain boundaries. Supplementary Table 1 shows the detailed composition of sulfide inclusions and their phases.

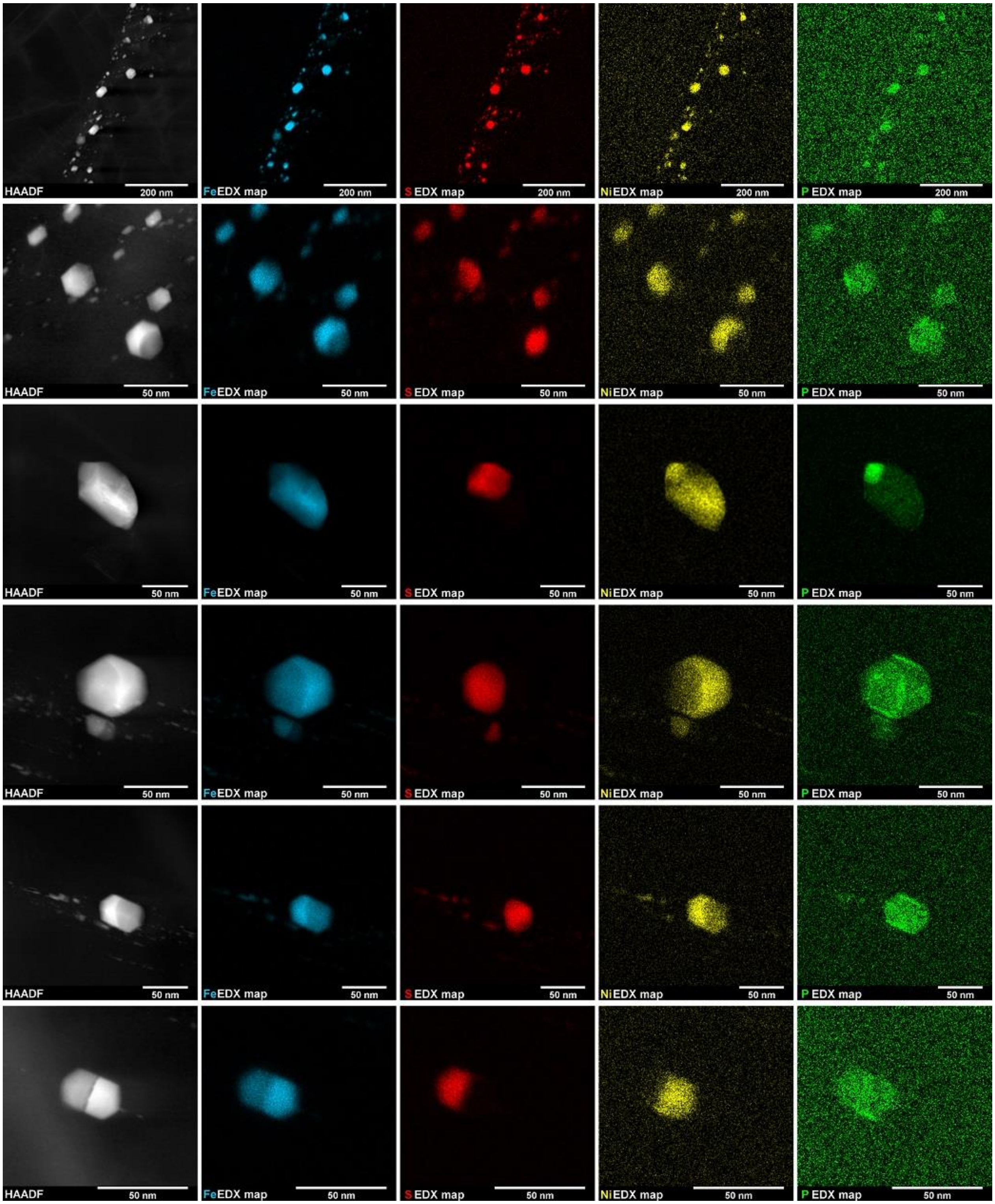

Supplementary Figure 4- HAADF STEM image and Fe, S, Ni, and P chemical (EDX) maps for several iron-sulfur type inclusions in diamonds.

Supplementary Table 1- Composition of Fe-S-bearing sulfides and their phases in atomic percent as measured by EDX.

Minerals	**Fe**	**Ni**	**S**	**P**	**Cr**
Kamacite	94.5%	5.5%	-	-	-
Troilite	50.4%	-	49.6%	(<0.7%)*	(<0.2%)*
Schreibersite	52.2%	22.3%	-	25.4%	-
$(Fe,Ni)_3(S,P)$	69.6%	5.1%	22.2%	3.16%	(<0.3%)**

*Due to the Cr and P enrichment in inclusion-diamond grain boundaries, the precise content of these elements in troilite could not be measured.

** Cr has been detected in inclusions with the composition between 0.1 and 0.3 atomic%.

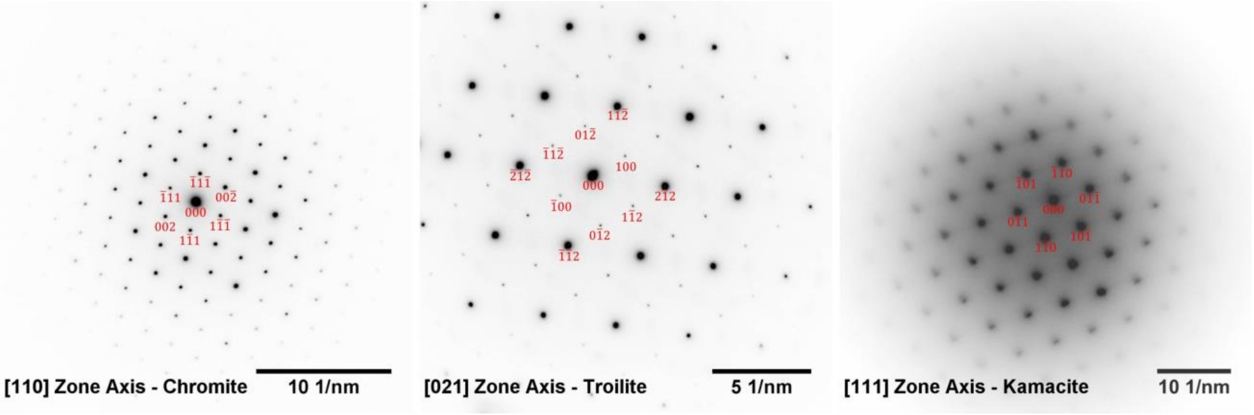

Supplementary Figure 5- From left to righ: SAED pattern of [110] zone axis in chromite, SAED pattern of [021] = [$\bar{2}4\bar{2}4$] zone axis in troilite, and nano-diffraction pattern of [111] zone axis in kamacite.

The composition and morphology of the inclusions suggest that they crystallized as single iron-rich S- and P-bearing phase. There are two known iron sulfide phases with higher iron concentration than troilite (FeS) namely: Fe_3S_2 and Fe_3S which crystallize above 14 GPa[1] and 21 GPa[2] respectively. To identify the parental phase of these inclusions we acquired tilt series from the inclusion veins and reconstructed their 3D shapes distinguishing the uncut and complete inclusions in depth of the samples from ones which were cut during the sample preparation. We have then acquired EDX maps from the same regions to quantify the elemental concentration in these inclusions. A total of 29 uncut inclusions were studied which were large enough to have high EDX counts for quantification and were not overlapping with other inclusions. We find that the bulk composition of the inclusions is $(Fe,Ni)_3(S,P)$ as shown in Table 1. The value (variability among 29 grains) of (Fe+Ni)/(S+P) is 2.98, with a standard deviation of 12%. The latter is probably a consequence of shadowing and absorption from the diamond matrix and multiple main phases in each inclusion which were randomly oriented in the matrix.

Fe_3S is stable above 21 GPa whereas (Fe, Ni)$_3$P is stable from ambient condition up to at least 50 GPa. Therefore the minimum pressure for stability and formation of their solid solution depends on the phosphorous content. As shown in Supplementary Fig. 6[3], for x lower than 0.2 in $Fe_3(S_{1-x},P_x)$, this solid solution can only form above 21 GPa. This substantiates the breakdown of the (Fe,Ni)$_3$(S,P) inclusion to stable lower-pressure polymorphs: troilite, kamacite, and schreibersite.

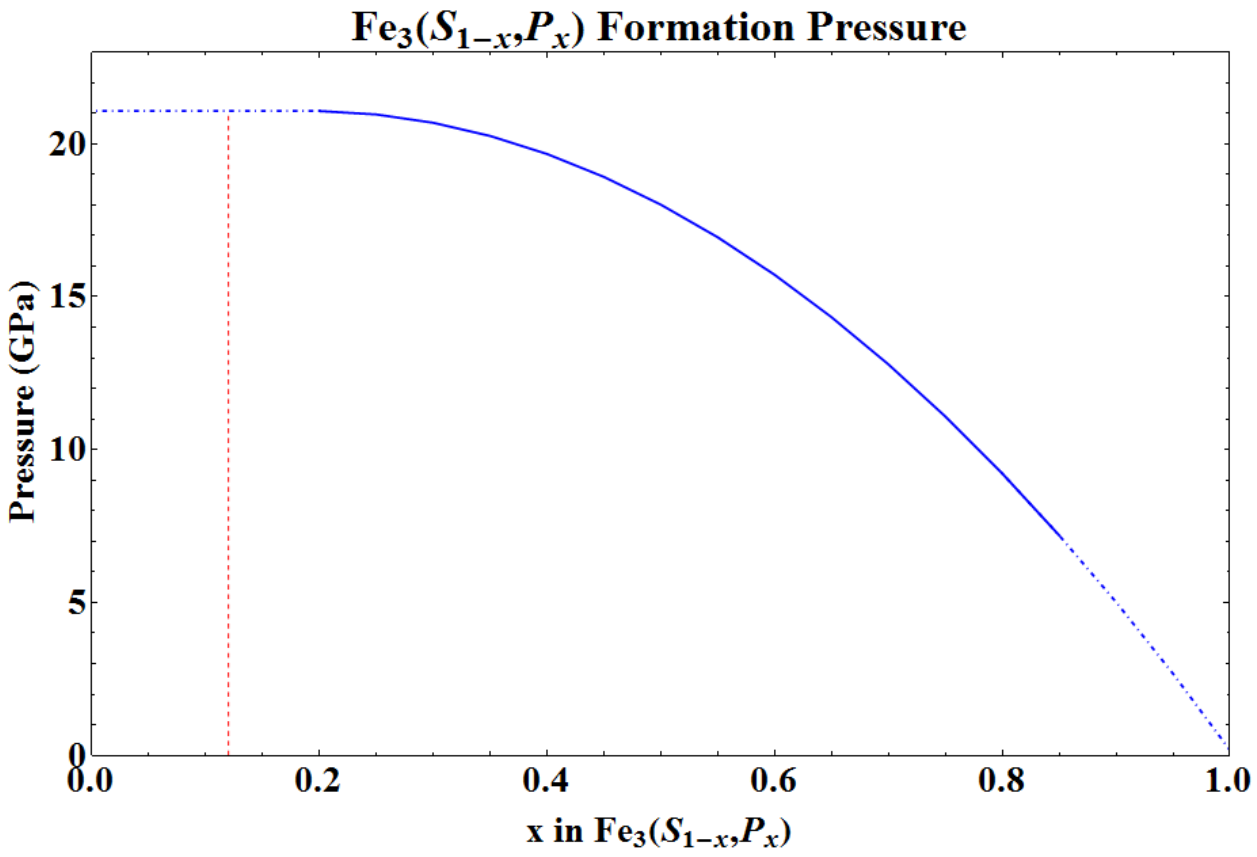

Supplementary Figure 6- The figure shows the lower pressure limit for $Fe_3(S_{1-x},P_x)$ formation versus the phosphorous concentration. For x equal or less than 0.2, this phase only forms above 21 GPa (after REF 3).

The Ni content can also affect the formation pressure of (Fe,Ni)$_3$(S,P) phase. Ni_3S forms above 5.1 GPa[4]. However, experimental data for the formation pressure of (Fe, Ni)$_3$S solid solution is not available. Therefore, we have used two end-members, Fe_3S and Ni_3S, to linearly approximate the variation of formation pressure for the $(Fe_{1-x}, Ni_x)_3S$ phase (Supplementary Fig. 7). For Ni/(Fe+Ni) = 0.07 this yields the formation pressure of 19.9 GPa (Supplementary Fig. 7). Considering that the real variation of formation pressure for such systems are often concaved downward (negative second derivative) as schematically shown in Supplementary Fig. 7, the value obtained from the linear approximation indicates the lower limit of the formation pressure for this composition.

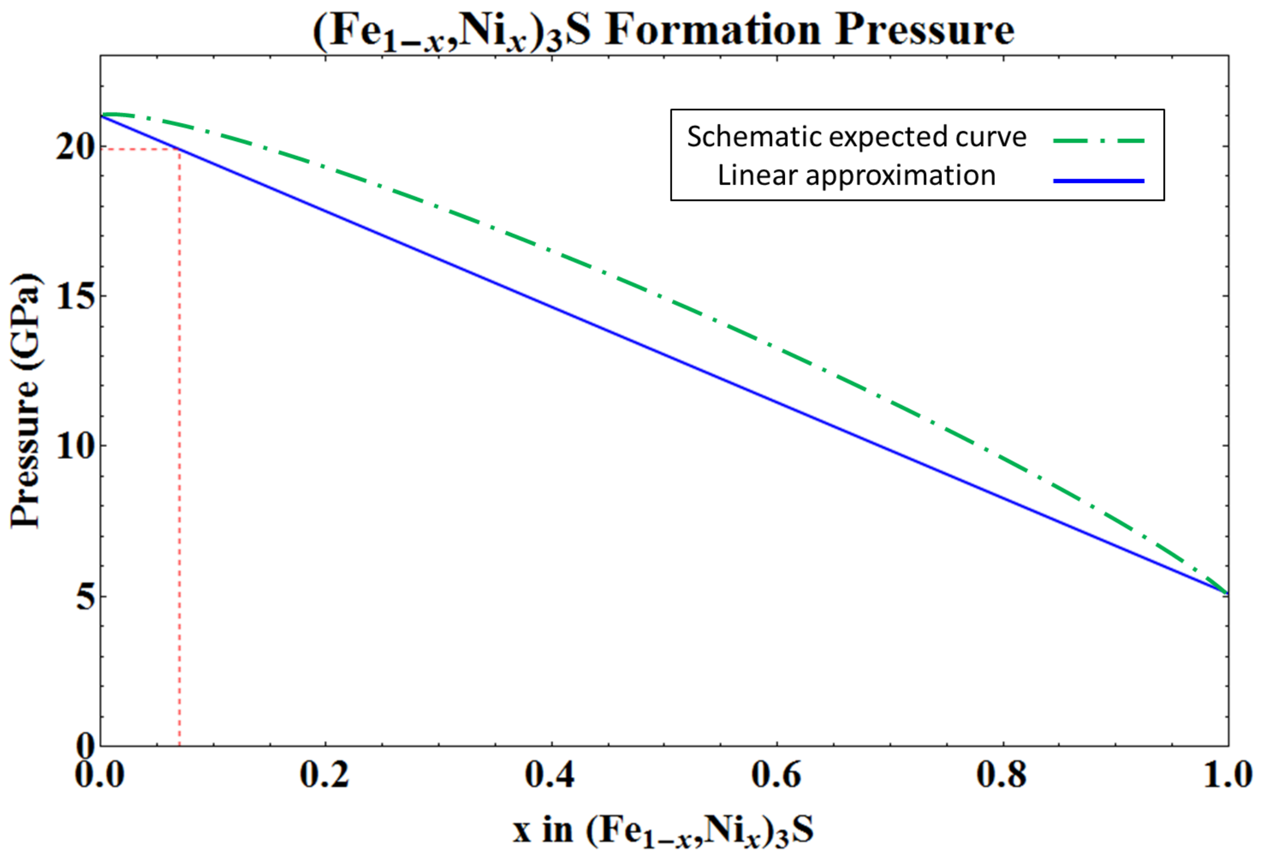

Supplementary Figure 7- Blue line shows the linear approximation for the formation pressure of $(Fe_{1-x}, Ni_x)_3S$ phase with respect to x. The green dashed line indicates the schematic shape of the expected curve for the plot. Red dashed line indicates the composition corresponding to the iron-sulfur inclusion studied here.

These sulfide inclusions could be syngenetic (i.e. forming at the same time as diamond), or protogenetic (i.e. forming before their encapsulation in diamond)[5]. Since these inclusions form trails in the diamond matrix, they could not have been subjected to a significant convection before diamond formation and their encapsulation. This supports the hypothesis that the diamond formed at the same time as the inclusions, or at least that the diamond were formed in the aftermath of inclusion formation, at the same place and condition that the inclusions had formed, namely in static high pressure condition of at least 20 GPa inside a planetary body.

Supplementary Note 3: Chromite and phosphate inclusions

Chromite Cr_2FeO_4 cation composition (excluding oxygen) is 64 atomic% Cr, 33.4 atomic% Fe, 1.8 atomic% Zn and 0.8 atomic% V. Electron diffraction confirms the spinel structure of chromite grains where Cr^{3+} and Fe^{2+} occupy octahedral and tetrahedral sites respectively (Supplementary Fig. 5 and 8). Zn is expected to substitute Fe in tetrahedral sites (as in zincochromite Cr_2ZnO_4) and V would occupy octahedral sites (as in coulsonite V_2FeO_4) like Cr. The (Cr+V)/(Fe+Zn) ratio is ~1.85. The slight deviation from the ratio of two could arise from

measurement errors as well as presence of Fe^{3+} in low concentration which would substitute Cr in octahedral sites (as in magnetite Fe_3O_4).

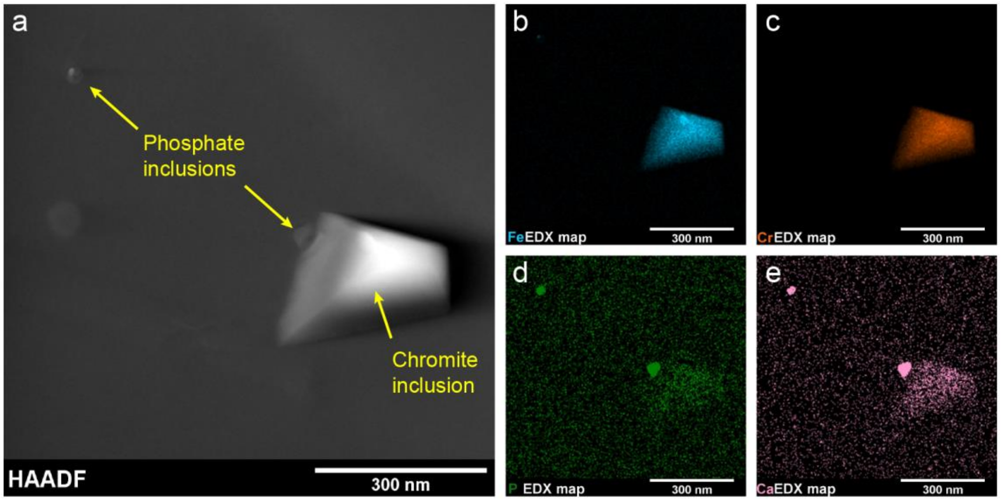

Supplementary Figure 8- a) HAADF STEM image and b) Fe, c) Cr, d) P, and e) Ca chemical (EDX) maps showing chromite and phosphate inclusions. At the top left of the HAADF image, bright and dark contrasts in the phosphate inclusion correspond to iron-rich and iron-poor regions. The slight bright contrast of P and Ca EDX maps in the chromite grain is the result of high background counts in that region (due to high density) and there is not actual P or Ca peak in chromite spectrum.

The phosphate inclusions are too small (~20 nm) and overlapping with other phases (Supplementary Fig. 8). Therefore we were not able to characterize them by electron diffraction. Although they are all Ca-Fe phosphate, they do not have uniform composition. Three of these inclusions were found close to one chromite inclusion, as is the case of iron meteorites. Two out of three were rich in Na and they contained low concentration of K. One of these phosphate inclusions has iron-rich and iron-poor parts.

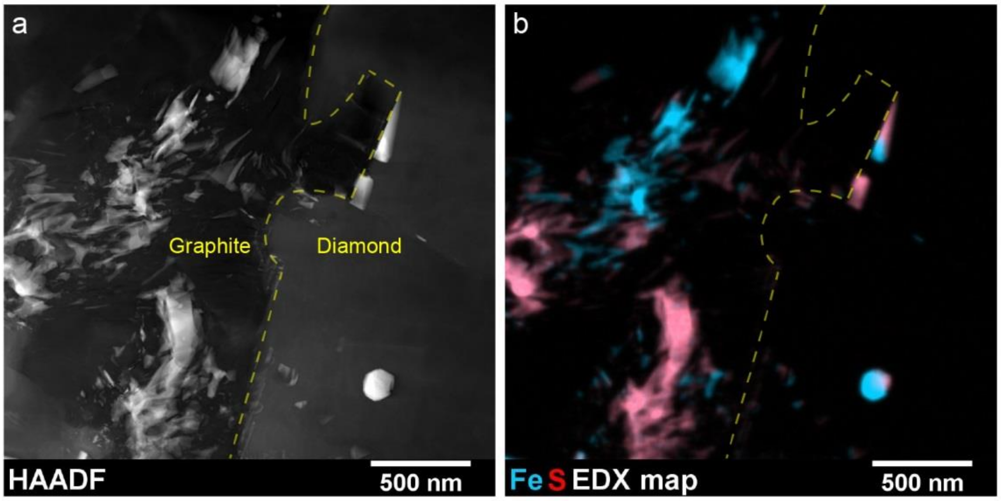

Supplementary Figure 9- a) HAADF STEM image and b) Fe-S chemical (EDX) map of inclusions in diamond and neighboring graphite. The dashed line shows the boundary between the two carbon polymorphs. We observe well-defined polyhedral shapes of inclusions in diamond in contrast with dispersed shape of inclusions in graphite. Separation of kamacite and troilite in graphite is also an indication for melting of inclusions during the graphitization.

Supplementary Table 2- Composition of intact iron-sulfur, $(Fe, Ni)_3(S, P)$, inclusions obtained from EDX.

#Inclusion	Fe (at%)	S (at%)	Ni (at%)	P (at%)	(Fe+Ni)/(S+P)
1	70.42	21.55	4.69	3.34	3.02
2	71.46	20.57	4.91	3.07	3.23
3	66.84	22.07	5.73	5.36	2.65
4	67.8	23.2	5.19	3.8	2.70
5	71.2	21.21	4.66	2.94	3.14
6	69.81	22.31	5.34	2.54	3.02
7	69.83	22.15	5.15	2.88	3.00
8	66.65	25.25	4.91	3.2	2.52
9	73.21	19.43	4.57	2.8	3.50
10	67.85	22.04	5.46	4.65	2.75
11	68.9	23.74	4.33	3.03	2.74
12	71.56	20.16	5.37	2.92	3.33
13	67.31	25.23	5.04	2.42	2.62
14	69.8	22.19	5.19	2.81	3.00
15	67.1	25.16	5.08	2.67	2.59
16	73.07	19.58	4.83	2.52	3.52
17	72.99	19.45	4.19	3.38	3.38
18	74.22	18.29	4.78	2.72	3.76
19	74.38	18.73	4.13	2.76	3.65
20	63.68	25.23	6.18	4.91	2.32
21	64.13	26.91	5.99	2.98	2.35
22	70.04	20.21	6.36	3.4	3.23
23	69.42	21.94	5.82	2.82	3.04
24	70.83	21.44	4.46	3.28	3.04
25	70.19	22.43	4.38	3	2.93
26	69.96	22.23	4.71	3.11	2.95
27	68.22	24.65	4.61	2.52	2.68
28	69.18	23.96	4.21	2.65	2.76
29	67.41	21.88	7.62	3.09	3.00
**Average**	69.57	22.18	5.10	3.16	2.98
**Standard Deviation**	2.67	2.18	0.77	0.70	0.36

Supplementary References:

1. Fei, Y., Bertka, C. M. & Finger, L. W. High-Pressure Iron-Sulfur Compound, Fe3S2, and Melting Relations in the Fe-FeS System. *Science* **275,** 1621–1623 (1997).
2. Fei, Y., Li, J., Bertka, C. M. & Prewitt, C. T. Structure type and bulk modulus of Fe3S, a new iron-sulfur compound. *Am. Mineral.* **85,** 1830–1833 (2015).
3. Gu, T., Fei, Y., Wu, X. & Qin, S. Phase stabilities and spin transitions of Fe3(S1−xPx) at high pressure and its implications in meteorites. *Am. Mineral.* **101,** 205–210 (2016).
4. Urakawa, S., Matsubara, R., Katsura, T., Watanabe, T. & Kikegawa, T. Stability and bulk modulus of Ni3S, a new nickel sulfur compound, and the melting relations of the system Ni-NiS up to 10 GPa. *Am. Mineral.* **96,** 558–565 (2011).
5. Taylor, L. A., Anand, M. & Promprated, P. Diamonds and their inclusions: are the criteria for syngenesis valid. in *8th International Kimberlite Conference. Long Abstract, Victoria, Canada* (Citeseer, 2003).
